# Beyond community state types: vaginal microbial diversity and CST-IV signatures are associated with pregnancy in euploid embryo transfer cycles

**DOI:** 10.3389/fendo.2026.1881146

**Published:** 2026-09-29

**Authors:** Yaoyu Zou, Wenbi Zhang, Aibo Gao, Jing Fu, Yilun Sui

**Affiliations:** 1 Reproductive Medical Centre, Department of Obstetrics and Gynecology, Ruijin Hospital, Shanghai Jiao Tong University School of Medicine, Shanghai, China; 2 Shanghai Ji Ai Genetics & IVF Institute, Obstetrics & Gynecology Hospital, Fudan University, Shanghai, China; 3 Department of Endocrine and Metabolic Diseases, Shanghai Institute of Endocrine and Metabolic Diseases, Ruijin Hospital, Shanghai Jiao Tong University School of Medicine, Shanghai, China

**Keywords:** clinical pregnancy, CST, euploid embryo transfer, IVF, vaginal microbiome

## Abstract

**Background:**

Despite significant progress in assisted reproductive technology, in vitro fertilization (IVF) pregnancy rates remain suboptimal. Although the vaginal microbiome has been implicated in IVF outcomes, prior studies often overlooked embryonic aneuploidy, a major confounder. This study aimed to investigate the association between the vaginal microbiome and pregnancy outcomes in euploid embryo transfer cycles.

**Methods:**

This prospective study included 109 single euploid blastocyst transfer cycles. Upper vaginal swabs were collected on the transfer day and subjected to 16S rRNA gene sequencing. Microbial α- and β-diversity, community state types (CSTs), and differentially abundant taxa were compared between the clinical pregnancy (CP, n = 55) and non-clinical pregnancy (NCP, n = 54) groups.

**Results:**

The NCP group exhibited significantly higher vaginal microbial richness (Chao1, p = 0.029; ACE, p = 0.029) and distinct β-diversity compared with the CP group (R² = 0.049, p = 0.002), whereas CST distribution did not differ between groups. Within the non-Lactobacillus-dominant CST-IV subgroup, the CP group was relatively enriched in Bifidobacteriaceae (LDA = 5.0) with predicted fermentative pathways, whereas the NCP group showed higher relative abundances of Prevotella (LDA = −4.1), Dialister (LDA = −3.6), and Peptoniphilus (LDA = −2.3), accompanied by predicted tryptophan degradation and oxidative stress pathways.

**Discussion:**

These findings, derived mainly from computational prediction, suggest that fine-scale taxonomic and functional composition, rather than broad CST classification, may influence pregnancy outcomes following euploid embryo transfer, offering preliminary insights for microbiome-informed strategies to optimize reproductive outcomes. Future longitudinal studies incorporating shotgun metagenomics and metabolomics in independent cohorts are warranted to validate these preliminary observations and to clarify the dynamic association between vaginal microbiota and IVF pregnancy outcomes.

## 1 Introduction

Despite the ongoing advancements in assisted reproductive technology (ART), the clinical pregnancy rate in *in-vitro* fertilization (IVF) remains suboptimal, typically ranging between 50–60% (1, 2). In addition to well-recognized factors such as age, body mass index (BMI), ovarian reserve and duration of infertility, there is growing attention directed toward the role of the reproductive tract microbiota in influencing ART outcomes (3–5).

As the first-line defense of the reproductive tract, the vaginal microbiome exhibits site-specific clinical relevance. Indeed, microbial dysbiosis of the female genital tract has been associated with a broad spectrum of gynecological disorders, including endometriosis, chronic endometritis, infertility, and even endometrial cancer (6), underscoring the far-reaching clinical significance of the gynecological microbial landscape. Recent comprehensive reviews have highlighted the critical role of vaginal dysbiosis in adverse reproductive outcomes, including implantation failure and spontaneous abortion (7). Its noninvasive accessibility (8), low risk of microbial contamination, and potential crosstalk with the uterus and ovarian microenvironment make it a pragmatic biomarker candidate. The upper reproductive tract, once considered sterile, is now recognized to harbor a low-biomass microbiota in continuity with the vaginal canal, and vaginal sampling has been reported to serve as a reasonable surrogate of the uterine microbial environment (6); accordingly, non-*Lactobacillus*-dominated endometrial profiles have been linked to impaired receptivity and adverse pregnancy outcomes (9). In healthy women, the vaginal microbiota is usually dominated by *Lactobacillu*s, which maintain an acidic environment hostile to pathogens (6, 10), mainly through the production of lactic acid, hydrogen peroxide, and bacteriocins (6). However, the presence of non-*Lactobacillus* bacteria, such as *Gardnerella*, *Atopobium*, and *Prevotella*, may reflect a less stable microbial state (11). The vaginal microbiome can be categorized into five community groups based on the composition and dominant species of *Lactobacillus* present. Community state types (CST) I, II, III, and V are primarily characterized by *Lactobacillus crispatus*, *Lactobacillus gasseri*, *Lactobacillus iners*, and *Lactobacillus jensenii*, respectively (6, 10). In contrast, CST IV is classified as non-*Lactobacillus*-dominated and contains a diverse array of anaerobic bacteria (10), and exhibits distinct immune characteristics (7, 12). Beyond local effects, genital microbiota can interact with the gut microbiome to shape host hormonal homeostasis, as bacterial β-glucuronidases modulate enterohepatic estrogen recycling to alter circulating estrogen levels and further influence endometrial physiology (6, 13).

Recent studies using deep sequencing techniques to analyze the microbiota of microorganisms in IVF patients have revealed the association between the vaginal microbiota diversity, the abundance of specific microorganisms, and/or the CST types with critical reproductive outcomes. These outcomes include embryo implantation rate (14), clinical pregnancy (15), and the recurrent implantation failure (16). Microbiota-based screening or interventions may potentially improve outcomes and alleviate patient burden (17). Accumulating evidence indicates that reproductive microbial dysbiosis contributes to impaired endometrial receptivity and adverse pregnancy outcomes, with chronic endometritis representing a typical dysbiosis-related reproductive disorder (18). Relevant studies have demonstrated that effective antimicrobial and probiotic interventions can alleviate intrauterine inflammatory status and optimize reproductive outcomes, further supporting the decisive role of microbial homeostasis in IVF treatment (18). However, existing results remain inconsistent (19–21), leaving a gap in the diagnostic and therapeutic applications of microbiome research in this field, and further research is needed to clarify these relationships. In particular, the functional characteristics of specific CST subtypes, especially CST IV, in the context of IVF remain incompletely understood. Furthermore, a major limitation of most prior studies is the lack of control for embryo ploidy, given that embryonic aneuploidy is a leading cause of implantation failure and miscarriage (22). This limitation severely compromises the interpretation of the true impact of the microbiome on pregnancy outcomes and constitutes one of the key factors underlying inconsistencies in the conclusions of existing studies.

To accurately assess the impact of vaginal microbiota, the study focuses on IVF cycles that involve the transfer of euploid blastocysts identified by preimplantation genetic testing (PGT). By prospectively analyzing vaginal microbiota on the day of embryo transfer, we aim to explore its association with pregnancy outcomes, with particular emphasis on CST stratification and its functional implications. This approach may offer valuable insights for potential clinical applications in ART and help identify patients who could benefit from microbiota-targeted interventions.

## 2 Methods

### 2.1 Study design and population

This is a prospective cohort study conducted at a tertiary hospital-based reproductive medicine center, which performs more than 5,000 IVF cycles annually, including over 1,000 PGT cycles, and also offers a full range of other assisted reproductive services, such as intrauterine insemination, fertility preservation, genetic counseling and testing, and reproductive endocrine care. The study included infertile patients who underwent preimplantation genetic testing for aneuploidy (PGT-A) at our center between April 2021 and October 2022. To mitigate the influence of uterine, immunological, and advanced maternal age factors on transplantation outcomes, only patients undergoing PGT-A due to a single previous adverse pregnancy history caused by embryonic chromosomal abnormality were included. All patients met the following criteria: 1) aged < 40 years; 2) received single embryo transfer with a blastocyst graded 5BC or higher (23). The exclusion criteria were as follows: 1) patients who had recurrent spontaneous abortion or recurrent implantation failure; 2) patients with premature ovarian insufficiency (AMH < 1.1 ng/ml and FSH > 12 mIU/ml), abnormal uterine cavity, endometriosis American Fertility Score III/IV, abnormal reproductive immunological findings, intrauterine adhesions as detected by hysteroscope, or system diseases; 3) those testing positive for mold spores or trichomonas in vaginal secretion examination and subjectively reported abnormal vaginal symptoms such as vaginal itching within 7 days before embryo transfer; 4) those having embryos underwent double vitrification or a second biopsy. Besides, the subjects should not receive antibiotics, probiotic use or vaginal medication within 7 days, or cervical treatment or flushing within 5 days of embryo transfer. The patient recruitment process and the application of the predefined inclusion and exclusion criteria are summarized in **Figure 1**.

**Figure 1:**
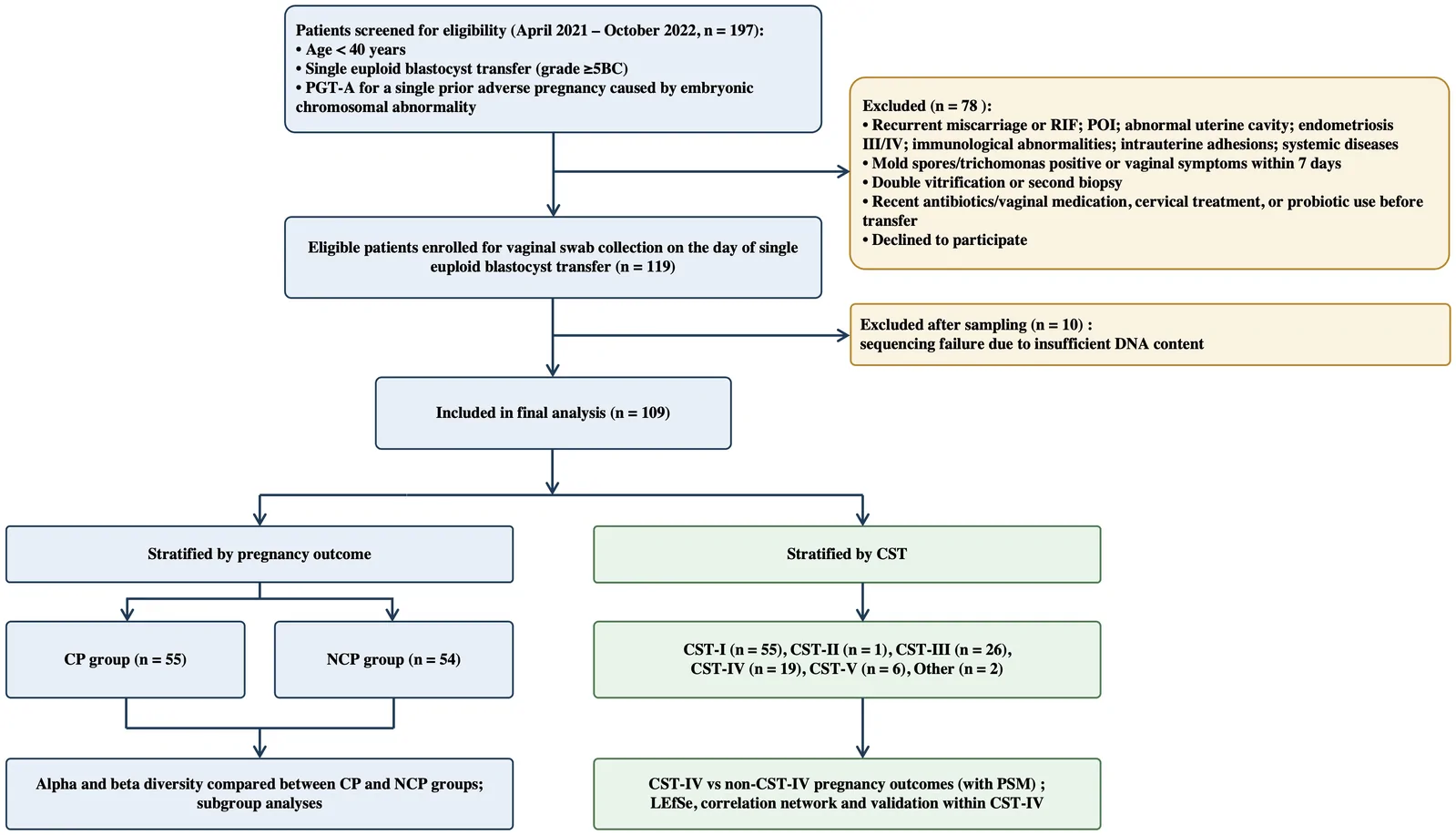
Flow chart describing the patient recruitment. The predefined inclusion and exclusion criteria and the corresponding numbers of participants at each stage are shown. PGT-A, preimplantation genetic testing for aneuploidy; RIF, recurrent implantation failure; POI, premature ovarian insufficiency; PSM, propensity score matching; CST, community state type; CP, clinical pregnancy; NCP, non-clinical pregnancy.

All participants provided their written informed consent. The study was approved by the ethics committee of the Shanghai JiAi Genetics & IVF Institute, with the reference number JIAI-E2020-022, and was granted clearance on November 17th, 2020. Additionally, this trial was registered at www.clinicaltrials.gov with registration number NCT06296615. This prospective observational study is reported in accordance with the Strengthening the Reporting of Observational Studies in Epidemiology (STROBE) guideline from the Enhancing the Quality and Transparency of health Research (EQUATOR) Network.

### 2.2 Embryological laboratory phase, PGT testing, and pregnancy outcome examination

The embryologists carried out oocyte retrieval, denudation, and intracytoplasmic sperm injection according to the standard clinical and laboratory procedures (24). Embryos were cultured in GIVF, G1, and G2 media (Vitrolife, Sweden) under conditions of 6% CO_2_ and 5% O_2_ at 37°C. Trophectoderm biopsy and cryopreservation were performed at the blastocyst stage, with biopsies subsequently analyzed for ploidy status using a next generation sequencing platform, following genetic laboratory guidelines (25). The transfer of a single blastocyst was executed once the endometrial thickness achieved 8 mm, subsequent to hormone replacement treatment using progesterone via intramuscular injection. CP was confirmed by ultrasonographic visualization of an intrauterine gestational sac at 7 weeks’ gestation. The NCP group comprised patients in whom no fetal heartbeat was detectable by ultrasound at this gestational age.

### 2.3 Vaginal samples collection

Upper vaginal samples were obtained on the day of embryo transfer under speculum visualization by trained obstetricians, prior to the transfer procedure. In brief, a sterile sample collection kit (Gongdong Co., Ltd., Taizhou, China: Universal Transport Medium for Viruses, Chlamydia, Mycoplasma and Ureaplasma) with a separated package was used in the sample collection. The kit comprised a long-handle cotton swab attached to a sealed cap and inserted in a collection tube. Care was taken during sampling to avoid contamination from the vulva, the speculum, or the physician’s gloves. And samples were promptly placed on ice and subsequently stored in a -80°C freezer within 20 minutes.

### 2.4 DNA extraction and PCR amplification

The total microbial genomic DNA was extracted from participant samples using the E.Z.N.A bacterial DNA Kit (Omega Bio-tek, Norcross, GA, U.S.). The DNA’s quality and concentration were evaluated through 1.0% agarose gel electrophoresis and kept at -80 °C. The hypervariable V3-V4 region of the bacterial 16S rRNA gene was amplified with the primers 319F and 806R (26). Each sample was amplified in triplicate PCR reactions to minimize amplification bias. PCR products were pooled, purified, quantified using the Quantus™ Fluorometer (Promega, USA).

### 2.5 16S rRNA gene sequencing

DNA products from each sample were quantified, and an equimolar pooling of the DNA was performed to ensure balanced representation of each sample in the subsequent library construction. The DNA library was then prepared using the NEBNext^®^ Ultra™ DNA Library Prep Kit (New England Biolabs, USA) following the manufacturer’s detailed protocol, which includes steps for end repair, A-tailing, adapter ligation, and enrichment of the library through PCR. The prepared libraries were then quality-checked and quantified before proceeding to sequencing. Paired-end sequencing was carried out on the Illumina MiSeq PE300 platform or NovaSeq PE250 paired-end sequencing platform (Illumina, USA), according to sequencing batch allocation.

### 2.6 Data processing

Raw sequencing data underwent rigorous quality control using fastp version 0.19.6 (26, 27), which was employed to eliminate low-quality sequences and adapters. Subsequently, paired-end reads were merged and filtered using FLASH version 1.2.7 (28) to ensure data integrity and accuracy. The filtering criteria were as follows: firstly, a sliding window of 50 base pairs (bp) was employed and reads with an average quality score below 20 within this window were truncated at 300 bp. Reads that fell short of 50 bp in length and those with ambiguous characters were discarded. Secondly, only sequences with an overlap exceeding 10 bp were assembled, thereby ensuring the reliability of the assembly process, and any reads that could not be assembled were discarded. Finally, samples were distinguished through precise barcode matching and allowing for two-base mismatches in primers, with adjustments made to the sequence orientation accordingly. Following this screening and organization, reads were grouped into operational taxonomic units (OTUs) using UPARSE 7.1 (29, 30) on the basis of a sequence similarity threshold greater than 97% and a confidence level below 0.7. This threshold was selected to ensure comparability with previous large-scale vaginal microbiome studies (31–33) and was deemed sufficient for resolving the genus- and family-level signatures central to our research questions. Representative sequences for each OTU were extracted for further analysis. These sequences were then taxonomically assigned using the SILVA database (release 138) as the reference. A bootstrap confidence threshold of 0.7 was applied to filter reliable taxonomic assignments. For OTUs annotated to the genus level but lacking confirmed species names, the corresponding representative sequences were further subjected to BLAST alignment in the NCBI database (34, 35). The species with the best hit was adopted as the species name. If species-level identification remained uncertain, the genus name with the placeholder “sp.” was used as a placeholder for subsequent analyses.

In this research, a total of 5,047,760 paired-end reads, averaging of 46,309 reads per sample, with 426 base pairs in length, were generated. After quality control, clustering, and removal of OTUs with fewer than 3 reads or samples with fewer than 2 reads, 794 high-quality OTU sequences were obtained.

### 2.7 Real-time PCR

Quantitative real-time PCR was performed using ChamQ SYBR color real-time PCR Master Mix (Vazyme, Q411-02, China) on a QuantStudio Dx system (Thermo Fisher Scientific Inc., USA). Primers targeting the universal bacterial 16S rRNA gene, together with species-specific primers for *Bifidobacterium bifidum* (NR_044771.1), *Prevotella bivia* (NR_113096.1), and *Dialister micraerophilus* (NR_041895.1) were used for amplification. Primer sequences were provided in **Supplementary Table 1**. The abundance of each bacterial species was normalized to the universal bacterial 16S rRNA gene, and relative bacterial abundance was calculated using the comparative Ct method (2^-ΔCt).

### 2.8 Data and statistical analysis

*A priori* sample size calculation was performed using PASS software (version 15.0) before participant recruitment. The calculation was based on the primary Chao1 richness index, comparing the CP and NCP groups. According to preliminary data and published literature, the expected mean difference in Chao1 richness between the two groups was assumed to be 3.0, with a pooled standard deviation of 4.5 (32). With a two-sided significance level (α) of 0.05 and a statistical power (1−β) of 0.90, the required sample size was estimated to be a total of 98 participants. Considering an anticipated dropout rate of 5%, the target enrollment was increased to 103 participants. Finally, 109 participants (55 in the CP group and 54 in the NCP group) were included in the analysis.

Baseline characteristics of the CP and NCP groups were compared using the χ^2^ test for categorical variables and the Wilcoxon rank–sum test for continuous variables, as the latter exhibited skewed distributions. Propensity score matching (PSM) using a 1:2 nearest neighbor matching algorithm with a caliper width of 0.2 was employed to compare the clinical outcomes between CST IV and non-CST IV groups, adjusting for age, BMI, endometrial thickness, AFC, and blastocyst grade. Additionally, the distribution of CST types between the CP and NCP groups, as well as clinical outcomes between patients with CST IV and those with non-CST IV profiles, were compared using χ^2^ tests or conditional logistic regression. All analyses involving clinical and demographic data were performed in SPSS version 25.0 (SPSS Inc., Chicago, IL, USA). Statistical analysis of the real-time PCR data was performed using GraphPad Prism 9 (GraphPad Software, San Diego, CA, USA). Data were presented as the mean ± standard error of the mean. Differences between the CP and NCP groups were evaluated using an unpaired two-tailed Student’s *t*-test. A two-sided *P* value < 0.05 was considered statistically significant.

All other analyses were conducted using R-4.3.1 software. To eliminate bias introduced by uneven sequencing depth, all samples were rarefied to the minimum read count within the dataset prior to alpha and beta diversity analysis. Based on the obtained OTU abundance matrix, the vegan package (36) was utilized to calculate the Chao1, ACE, Shannon, and Simpson index for Alpha diversity was calculated as the effect size for each comparison. Pairwise comparisons across multiple subgroups were performed using Wilcoxon rank-sum tests, and *p* values were adjusted via Benjamini–Hochberg FDR correction to control false discovery rate. Beta diversity was assessed using non-metric multidimensional scaling (NMDS) analysis, and the results were visualized using ggplot2. The differences between the groups were analyzed using ANOSIM (37). Stacked bar chart was generated using ggplot2 package, with bars representing the relative abundance of genera stacked within each sample. The clustering heatmap was constructed based on the relative abundances of predominant species across samples. Species with the highest variance or differential abundance among CST groups were selected for visualization. Relative abundance data were log-transformed and standardized using Z-score normalization prior to clustering.

Differential bacterial taxa spanning phylum to genus were screened using LEfSe with the one-against-all strategy (38), with subgroup analyses restricted to CST-IV to avoid confounding by dominant CST-driven taxonomic differences and to target the subgroup with highest microbial heterogeneity and clinical relevance. All *p* values were corrected via the Benjamini–Hochberg false discovery rate method. Taxa with adjusted p < 0.05 and a linear discriminant analysis (LDA) effect size > 2 were considered significantly different (39). Candidate signature taxa distinguishing CP and NCP groups underwent additional validation with FDR-adjusted Wilcoxon rank-sum tests based on relative abundance. The core discriminatory genera *Bifidobacterium*, *Prevotella* and *Dialister* were quantified through real-time PCR to experimentally validate sequencing observations. For the selection of species in the correlation network analysis, a criterion of Spearman correlation coefficient |r| > 0.6 and *p* < 0.05 was applied. The resulting network was then visualized using Cytoscape software. Functional profiles of the microbial communities were predicted using PICRUSt2 against the KEGG Ortholog database. Differentially abundant metabolic pathways predicted by PICRUSt2 were also subjected to FDR correction.

## 3 Results

### 3.1 Study population and baseline characteristics

A total of 119 eligible patients were enrolled with the intention of providing vaginal swabs for bacterial analysis at the transfer of single euploid embryo. However, sequencing failed in 10 patients due to insufficient DNA content. No significant differences in baseline characteristics were observed between excluded and included patients (p > 0.05). Consequently, 109 patients were included in the final analysis (**Figure 1**), with an overall clinical pregnancy rate of 50.46%. The patients were subsequently classified into a clinical pregnancy (CP, N = 55) or non-clinical pregnancy (NCP, N = 54) group. There was no significant difference in the baseline characteristics between the two groups in terms of age, BMI, duration of infertility, endometrial thickness, or embryo quality (p > 0.05), confirming successful control of major confounding variables (**Table 1**).

**Table 1:** Baseline characteristics of the women who became pregnant and did not become pregnant.

Variable	CP (N = 55)	NCP (N = 54)	*p*-value
Age at oocyte retrieval (year)	32.00 (30.00, 36.00)	32.00 (29.00, 37.00)	0.122
BMI (kg/m^2^)	20.57 (19.36, 22.66)	21.84 (19.49, 23.77)	0.154
Duration of infertility (year)	3.00 (1.00, 4.00)	2.50 (1.00, 6.00)	0.943
Ovarian stimulation protocol			0.213
Short GnRH agonist protocol	24 (44.44%)	30 (55.56%)	
GnRH antagonist protocol	31 (56.36%)	24 (43.64%)	
No. of controlled ovarian stimulation cycles	1.00 (1.00, 2.00)	1.00 (1.00, 2.00)	0.502
No. of antral follicles	20.00 (12.00, 25.00)	15.50 (12.00, 22.25)	0.177
No. of retrieved oocytes	15.00 (11.00, 21.00)	14.00 (11.00, 17.00)	0.298
No. of day 3 available embryos	10.00 (7.00, 14.00)	8.00 (5.00, 10.00)	0.113
No. of available blastocysts	5.00 (3.00, 9.00)	5.00 (3.00, 7.00)	0.055
Age at transfer (year)	32.00 (31.00, 36.00)	32.00 (30.00, 37.00)	0.112
No. of transfer cycles	1.00 (1.00, 3.00)	1.00 (1.00, 3.00)	0.927
Endometrial thickness at transfer (mm)	9.00 (8.00, 10.00)	9.00 (8.00, 11.00)	0.501
Use of antibiotics in prior 3 months			0.329
Yes	10 (41.67%)	14 (58.33%)	
No	45 (52.94%)	40 (47.06%)	

Data are presented as Number (%) or Median (IQR).

*P* values were calculated using χ2 tests for categorical variables, or Mann-Whitney test for skewed distributed continuous variables. CP=clinical pregnancy; NCP=non-clinical pregnancy.

### 3.2 Diversity analyses of vaginal microbial communities

The rarefaction curves of OTU samples reached plateaus with increasing sequencing depth (**Figure 2A**), indicating that the current sequencing adequately captured the majority of microbial diversity within the samples. At the community level, Alpha diversity analysis revealed a significant difference in the richness of vaginal microbial communities between the CP and NCP groups. The NCP group exhibited significantly higher Chao1 richness (*p* = 0.029), *r* = -0.24, **Figure 2B**) and ACE richness (*p* = 0.029, *r* = -0.17, **Figure 2C**) compared to the CP group, suggesting greater bacterial richness in women who did not achieve clinical pregnancy. However, no significant differences were observed in the Shannon or Simpson diversity indices (**Figures 2D, E**), indicating that while the number of species differed between groups, their relative abundances and overall community evenness remained comparable. Subgroup analyses further confirmed that the NCP group maintained significantly higher alpha diversity in both thick (≥10 mm) (**Supplementary Figure S1A**) and thin (8–10 mm) endometrium subgroups (**Supplementary Figure S1B**). Higher Chao1 richness in the NCP group was observed among normal responders (*p* = 0.047, **Supplementary Figure S1C**) and first-time transfer patients (*p* = 0.018, **Supplementary Figure S1G**), but not among high responders (**Supplementary Figure S1D**) or those with repeated transfers (**Supplementary Figure S1H**). In patients without recent antibiotic use, the NCP group exhibited significantly higher ACE (*p* = 0.033) and Chao1 (*p* = 0.019) indices (**Supplementary Figure S1E**), while this association was not observed in patients with antibiotic use in prior 3 months (**Supplementary Figure S1F**). For embryo grade, the NCP group had significantly higher Chao1 (*p* = 0.008), Shannon (*p* = 0.031) and Simpson (*p* = 0.031) indices in the BB subgroup (**Supplementary Figure S1I**), with no difference in the BC subgroup (**Supplementary Figure S1J**). No significant differences were found in <3-year and ≥3-year infertility subgroups (**Supplementary Figures S1K, L**).

**Figure 2:**
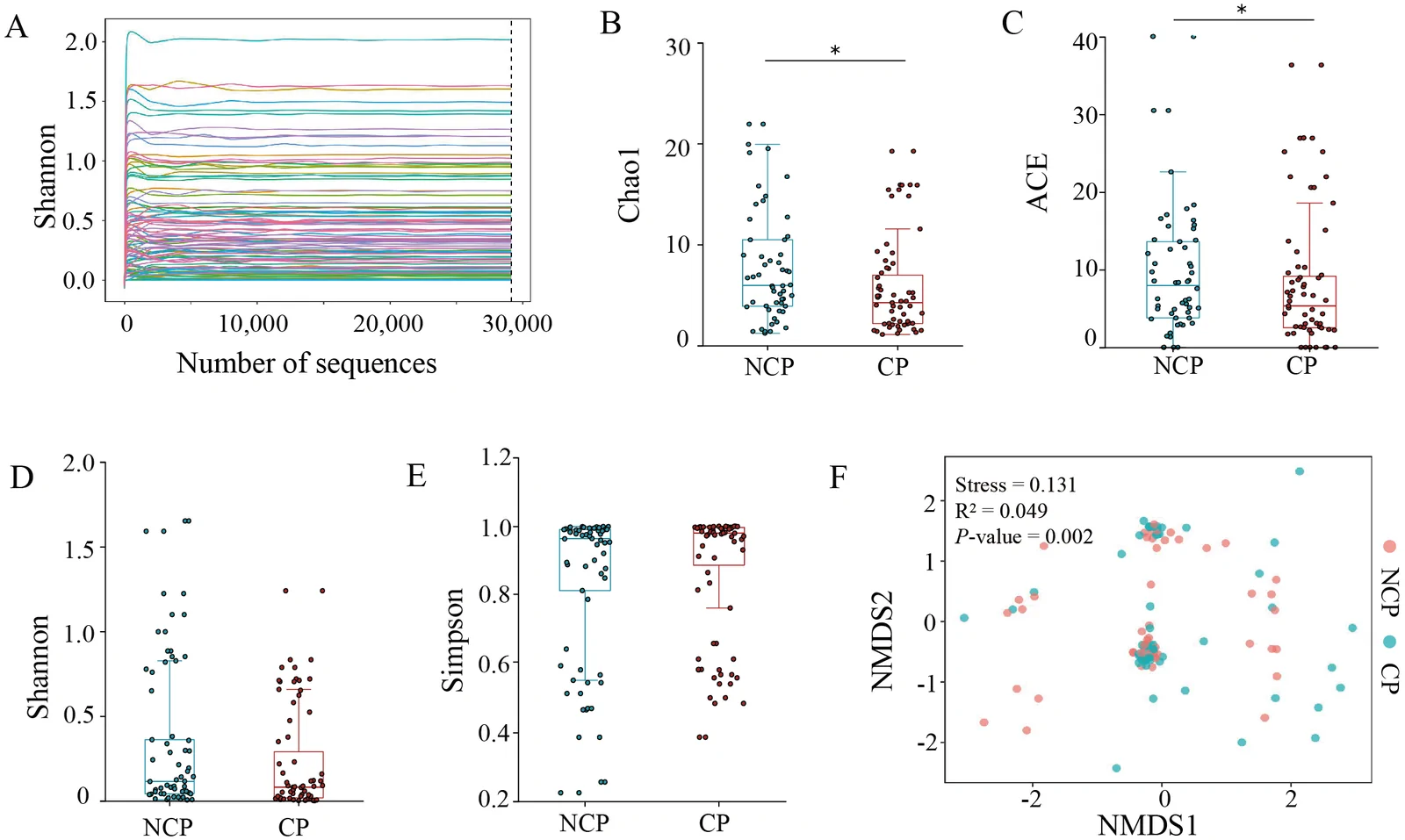
Alpha and Beta diversity of vaginal microbiota in the NCP and CP groups. Species rarefaction curve **(A)**, Chao1 index **(B)**, ACE index **(C)**, Shannon index **(D)**, Simpson index **(E)**, and NMDS analysis **(F)** of the samples from NCP and CP patients. * indicating significant differences between the two groups.

Beta-diversity analysis based on Bray–Curtis dissimilarity, visualized via NMDS, revealed a statistically significant but relatively modest separation between the CP and NCP groups (**Figure 2F**, ANOSIM, R^2^ = 0.049, *p* = 0.002), indicating that pregnancy outcome explains approximately 5% of the total variance in vaginal microbial community structure. Subgroup beta-diversity analyses across all stratification variables showed no significant separation between CP and NCP groups (all ANOSIM *p* > 0.05), indicating that the modest outcome-associated community shift observed in the overall cohort was not driven by any single clinical or physiological subgroup.

### 3.3 CST stratification and clinical outcomes

At the genus level, the vaginal microbiota of most patients was relatively uniform and was predominantly composed of *Lactobacillus* (**Figure 3A**). However, a distinct subgroup exhibited non-*Lactobacillus-*dominant profiles, primarily enriched in genera such as *Gardnerella*, *Atopobium*, *Bifidobacterium*, and *Streptococcus* (**Figure 3A**). Based on hierarchical clustering of species-level taxonomic profiles (**Figure 3B**), samples were classified into five community state types (CSTs) according to the criteria by Ravel et al. (10): CST-I (n = 55), CST-II (n = 1), CST-III (n = 26), CST-IV (n = 19), CST-V (n = 6), and an unclassifiable “Other” group (n = 2). Notably, the aforementioned non-*Lactobacillus*-dominant subgroup corresponds precisely to the CST-IV subgroup, which is characterized by high microbial diversity and the absence of a dominant *Lactobacillus* species.

**Figure 3:**
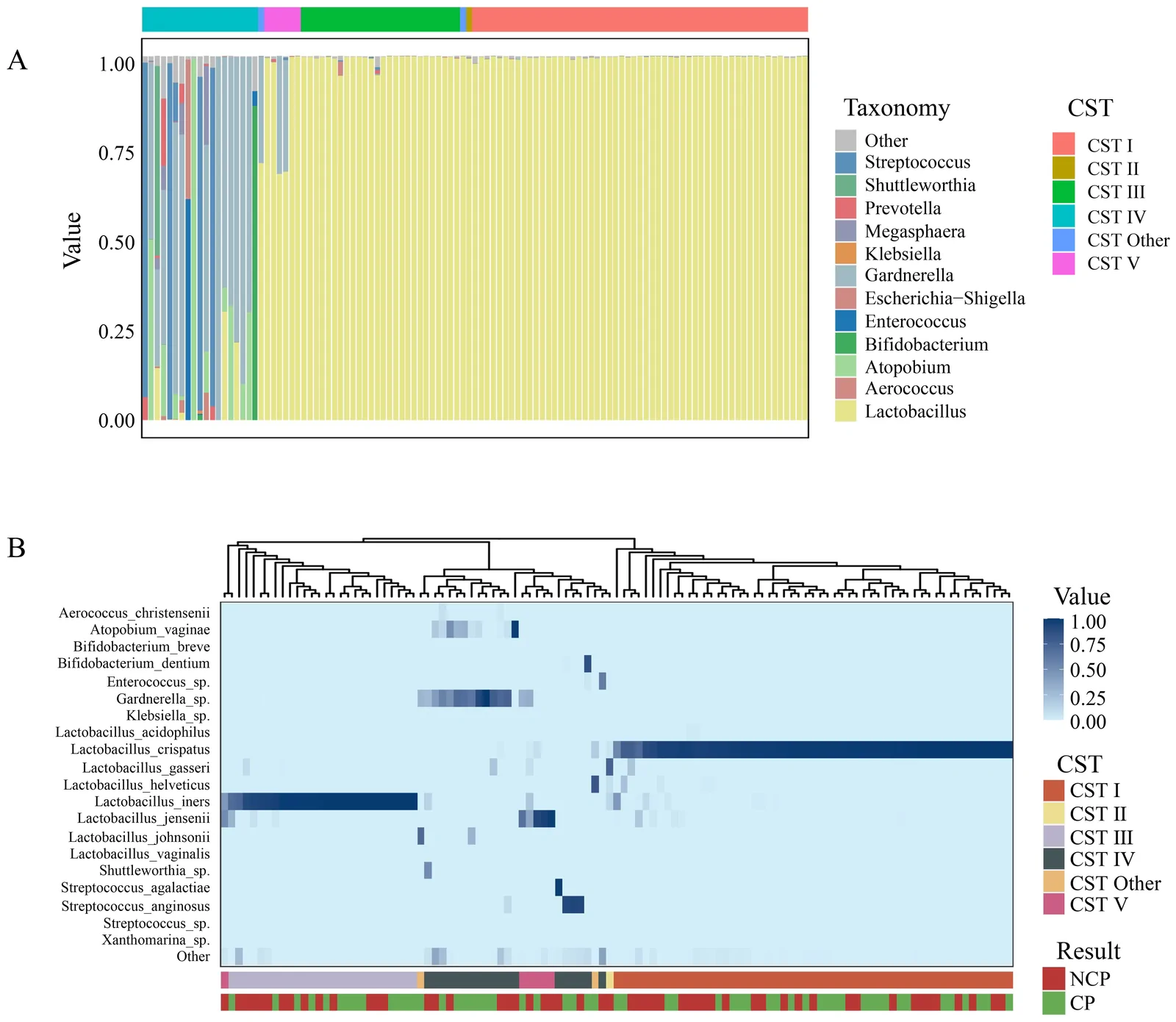
Composition of vaginal microbiota structure. **(A)** Stacked bar chart at the genus level depicting abundances exceeding 1%. **(B)** Hierarchical clustering heatmap illustrating the predominant species that vary among different CST types across samples. The blue color represents the relative abundance of bacterial communities. CST, community state type.

Given the limited sample sizes of CST-II and CST-V subgroups, subsequent diversity analyses focused on CST-I, CST-III, and CST-IV subgroups. Specifically, the CST-IV subgroup, characterized by the absence of *Lactobacillus* and enrichment of diverse anaerobes, exhibited significantly higher alpha diversity (Chao1, ACE, Shannon, and Simpson; all *p* < 0.05; **Figure 4**) compared to *lactobacillus*-dominant CST-I and CST-III subgroups, reflecting its greater compositional complexity and heterogeneity.

**Figure 4:**
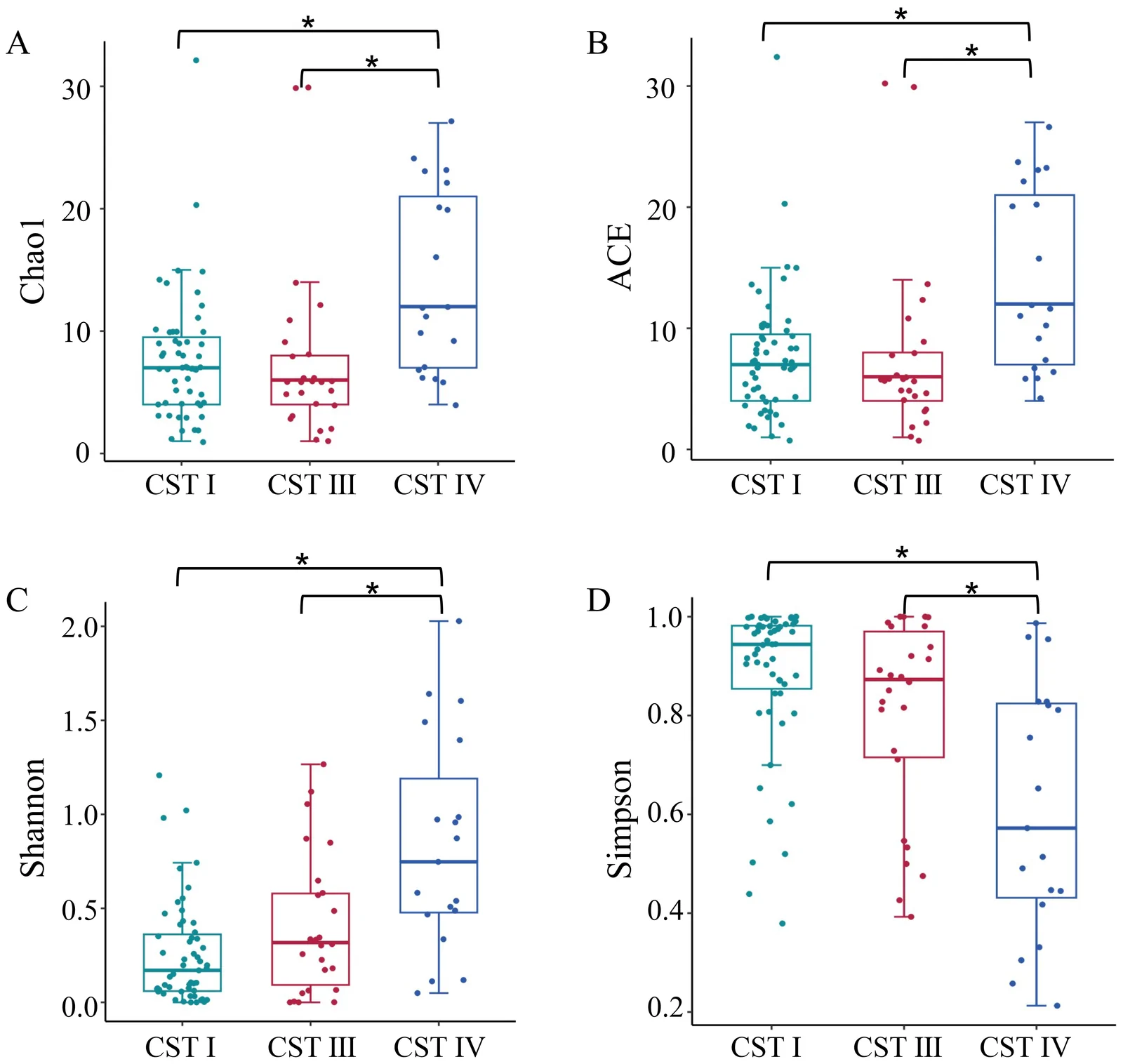
Alpha diversity of vaginal microbiota in CST subgroups. Chao1 index **(A)**, ACE index **(B)**, Shannon index **(C)**, Simpson index **(D)** of the samples from CST subgroups. * indicating significant differences between the two groups.

Despite the observed microbial differences, pregnancy outcomes including implantation, clinical pregnancy, ongoing pregnancy, and live birth following single euploid embryo transfer were comparable between CST-IV and non-CST-IV subgroups (**Table 2**). After adjusting for baseline confounders via PSM, clinical pregnancy rates remained non-significant between the two subgroups (**Table 2**; **Supplementary Table 2**). Moreover, the CST distribution did not differ between CP and NCP groups (p = 0.589, Cramer’s V = 0.099).

**Table 2:** Comparison of clinical pregnancy outcomes between CST IV and non-CST IV groups before and after propensity score matching. .

Variable	CST IV (n=19)	non-CST IV (n=90)	PSM matched non-CST IV (n=38, 1:2 matched)	Rate difference (95%CI) before PSM	Rate difference (95%CI) after PSM
Biochemical pregnancy rate	63.16% (12/19)	64.44% (58/90)	60.53% (23/38)	-1.29% (-25.1%, 22.6%)	2.63% (-24.1%, 29.3%)
Clinical pregnancy rate	47.37% (9/19)	51.11% (46/90)	55.26% (21/38)	-3.74% (-28.5%, 21.0%)	-7.89% (-35.4%, 19.6%)
Ongoing pregnancy rate	47.37% (9/19)	48.89% (44/90)	55.26% (21/38)	-1.52% (-26.2%, 23.2%)	-7.89% (-35.4%, 19.6%)
Live birth rate	47.37% (9/19)	47.78% (43/90)	50.00% (19/38)	-0.41% (-25.1%, 24.3%)	-2.63% (-30.1%, 24.9%)

Values are presented as percentage (events/total). Rate differences with 95% confidence intervals (CIs) are provided. Propensity score matching was performed at a 1:2 ratio (caliper width = 0.2), adjusting for age, BMI, endometrial thickness, AFC, and blastocyst grade. Pre-PSM P values were derived from Pearson’s χ^2^ test; post-PSM *p* values from conditional logistic regression. No statistically significant differences were observed in any pregnancy outcome (all *p* > 0.05).

### 3.4 Distinct vaginal microbial signatures in CST IV differentiate clinical pregnancy from non-clinical pregnancy

Linear discriminant analysis (39) scores calculated by LEfSe analysis were employed to evaluate the effect size of each microbial taxon on differentiating the pregnancy outcomes. The analysis spanned taxonomic levels from phylum to genus to identify differentially abundant bacteria between the CP and NCP groups within CST IV (**Figures 5A, B**). In the CP group, the family *Bifidobacteriaceae* (LDA = 5.0) was identified as the key bacterium. Conversely, the NCP group was characterized by three key genera: *Peptoniphilus* (LDA = −2.3), *Dialister* (LDA = −3.6), and *Prevotella* (LDA = −4.1); along with the family *Veillonellaceae* (LDA = −4.1). FDR-adjusted Wilcoxon tests confirmed significant intergroup differences in the abundance for these signature taxa. Bootstrap sensitivity analysis provided the following effect sizes and 95% confidence intervals: *Bifidobacteriaceae*, 32.67 (95% CI, 4.277–60.58); *Peptoniphilus*, −0.02, (95%CI, −0.053 to −0.002); *Veillonellaceae*, −0.23 (95%CI, −4.142 to 5.578); *Prevotella*, −3.15, (95%CI, −7.306 to 0.222); *Dialister* −0.64, (95%CI, −1.305 to −0.125) (**Supplementary Table 3**). Stratified LEfSe across clinical subgroups (**Supplementary Figures S2A-J**) confirmed that CP-associated *Bifidobacteriaceae* and NCP-associated *Dialister*, *Prevotella*, and *Veillonellaceae* signatures remained consistent in patients with thin endometrium (**Supplementary Figure S2A**), normal ovarian response (**Supplementary Figure S2C**), first transfer (**Supplementary Figure S2F**), no antibiotic exposure (**Supplementary Figure S2E**), BB-grade embryos (**Supplementary Figure S2G**), or infertility duration ≤3 years (**Supplementary Figure S2I**). In contrast, *Fusobacteriota* was uniquely enriched in NCP patients with infertility ≥3 years (**Supplementary Figure S2J**), whereas taxonomic differences between CP and NCP were attenuated among high responders (**Supplementary Figure S2D**) and BC-grade embryo recipients (**Supplementary Figure S2H**).

**Figure 5:**
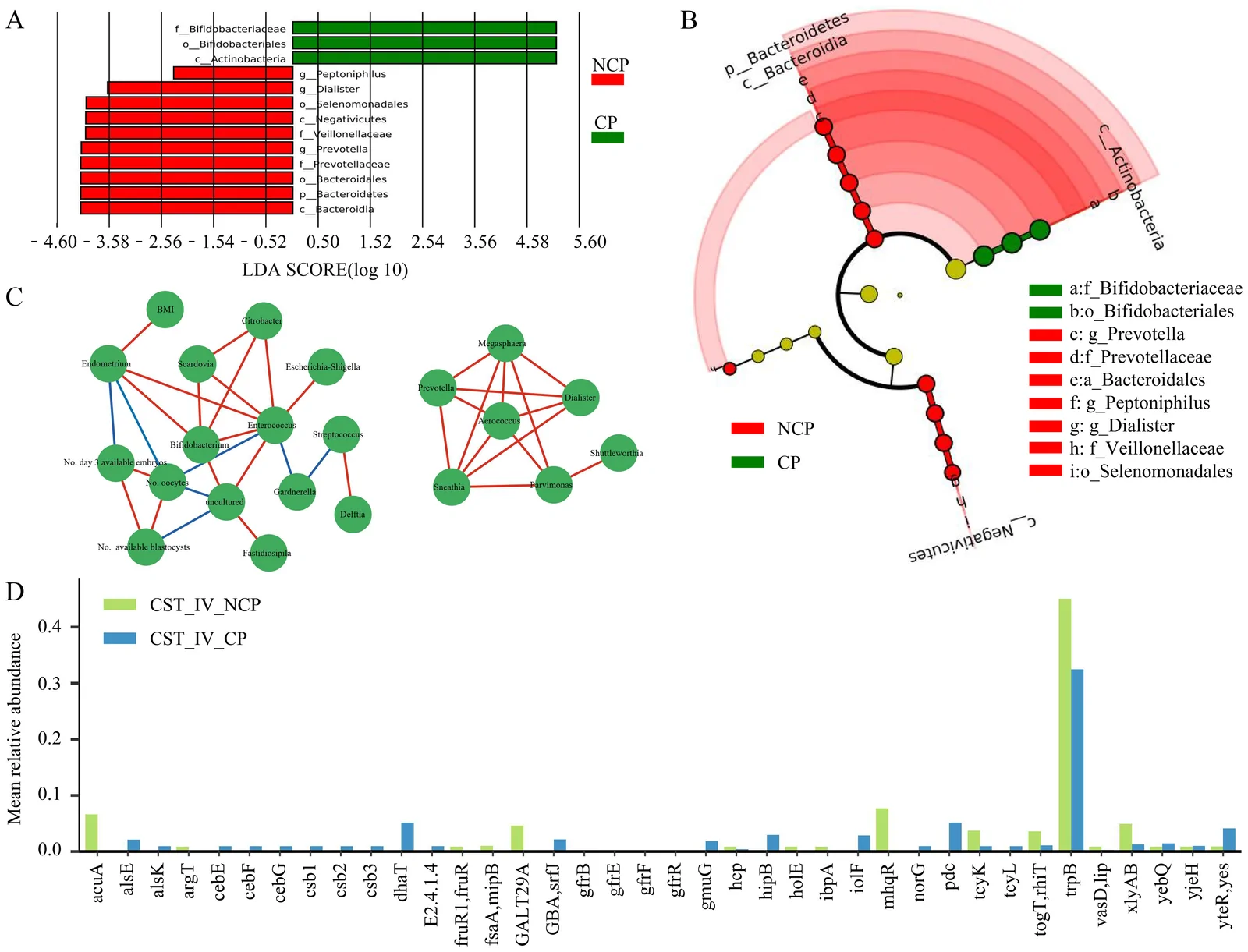
The association between vaginal microbial community with pregnancy outcome. **(A)** Linear discriminant analysis of the differentially abundant genera (LDA score >2), which indicated their contribution to group differentiation. **(B)** Cladogram illustrating the phylogenetic branches of microbial communities meeting the criteria for LDA scores. **(C)** Network analysis of correlations between basic sample indicators and genera with abundances exceeding 1% (p<0.05 and |r|≥0.6), edge color: red represents positive correlation, blue represents negative correlation. **(D)** Predicted functional profiles of CST IV-type bacteria in the NCP and CP groups based on PICRUSt2. LDA, linear discriminant analysis effect size.

To validate the sequencing findings, *Prevotella bivia* (40), *Dialister micraerophilus* (41), and *Bifidobacterium bifidum* (42, 43) were selected for species-level validation based on the LEfSe results and their biological plausibility reported in previous studies. Consistent with the sequencing results, the relative abundance of *Prevotella bivia* was significantly higher in the NCP group than in the CP group (**Supplementary Figure S3A**, *p* = 0.042), whereas no significant difference was observed for *Dialister micraerophilus* (**Supplementary Figure S3B**, *p* = 0.384). In contrast, *Bifidobacterium bifidum* showed a significantly lower relative abundance in the NCP group than in the CP group (**Supplementary Figure S3C**, *p* = 0.031).

Correlation network analysis further revealed a bifurcated structure, anaerobic genera including *Prevotella* and *Dialister* which are associated with NCP formed a tightly connected module, whereas *Bifidobacterium* correlated with positive pregnancy outcome and showed a direct positive association with endometrial thickness at embryo transfer (**Figure 5C**). Computational functional prediction using PICRUSt2 suggested that NCP samples may exhibit a higher abundance of tryptophan degradation (trpB) and oxidative stress response regulation (mhqR). In contrast, CP samples were enriched in pyruvate decarboxylase (pdc), which plays a key role in fermentation-related metabolic pathways (**Figure 5D**). While these predictions hint at potential mechanisms involving oxidative stress and energy metabolism, they represent inferred metabolic potential rather than direct evidence of microbial activity.

Collectively, these findings suggest that even within the same CST-IV classification, pregnancy outcome is associated with fine-scale taxonomic composition and predicted metabolic potential. This identifies a distinct microbial profile characterized by specific functional signatures that may be permissive for implantation despite the absence of dominant *lactobacilli*.

## 4 Discussion

This study employed a rigorously controlled cohort of single euploid blastocyst transfers with well-matched baseline characteristics. This design allowed us to isolate the specific contribution of the vaginal microbiome to embryo implantation while controlling for embryonic aneuploidy and minimizing major clinical confounders as sources of variability. Our results demonstrate that although the NCP group exhibited significantly higher richness of vaginal microbial communities, broad CST classification is not associated with clinical pregnancy outcomes following euploid embryo transfer. This finding is supported by recent insights suggesting that the reproductive tract microbiome should not be viewed merely through the binary lens of “*Lactobacillus* dominance” versus “dysbiosis” (7). Specifically, women with non-*Lactobacillus*-dominant CST-IV achieved pregnancy outcomes comparable to those with *Lactobacillus*-dominant CSTs, challenging the prevailing view in reproductive microbiology that non-*Lactobacillus*-dominant communities confer inferior reproductive success (7, 16, 21, 44–47). The results suggested that successful pregnancy was not determined by the broad CST classification but rather stratified by refined microbial characteristics. Subgroup analysis within CST-IV further revealed that successful pregnancy was associated with *Bifidobacteriaceae* enrichment and inferred fermentative pathways, whereas failed cases were associated with higher relative abundances of *Prevotella*, *Dialister*, and *Peptoniphilus*, alongside predicted tryptophan degradation and oxidative stress response pathways (**Figure 6**). These results suggest that reproductive competence may not rely solely on *Lactobacillus* dominance, but could potentially involve commensals that are predicted to contribute to a permissive microenvironment for euploid embryos. However, as these functional profiles are computationally inferred from 16S rRNA sequencing data, they represent preliminary hypotheses rather than confirmed metabolic activities, warranting validation in independent cohorts and through metagenomic or metabolomic approaches.

**Figure 6:**
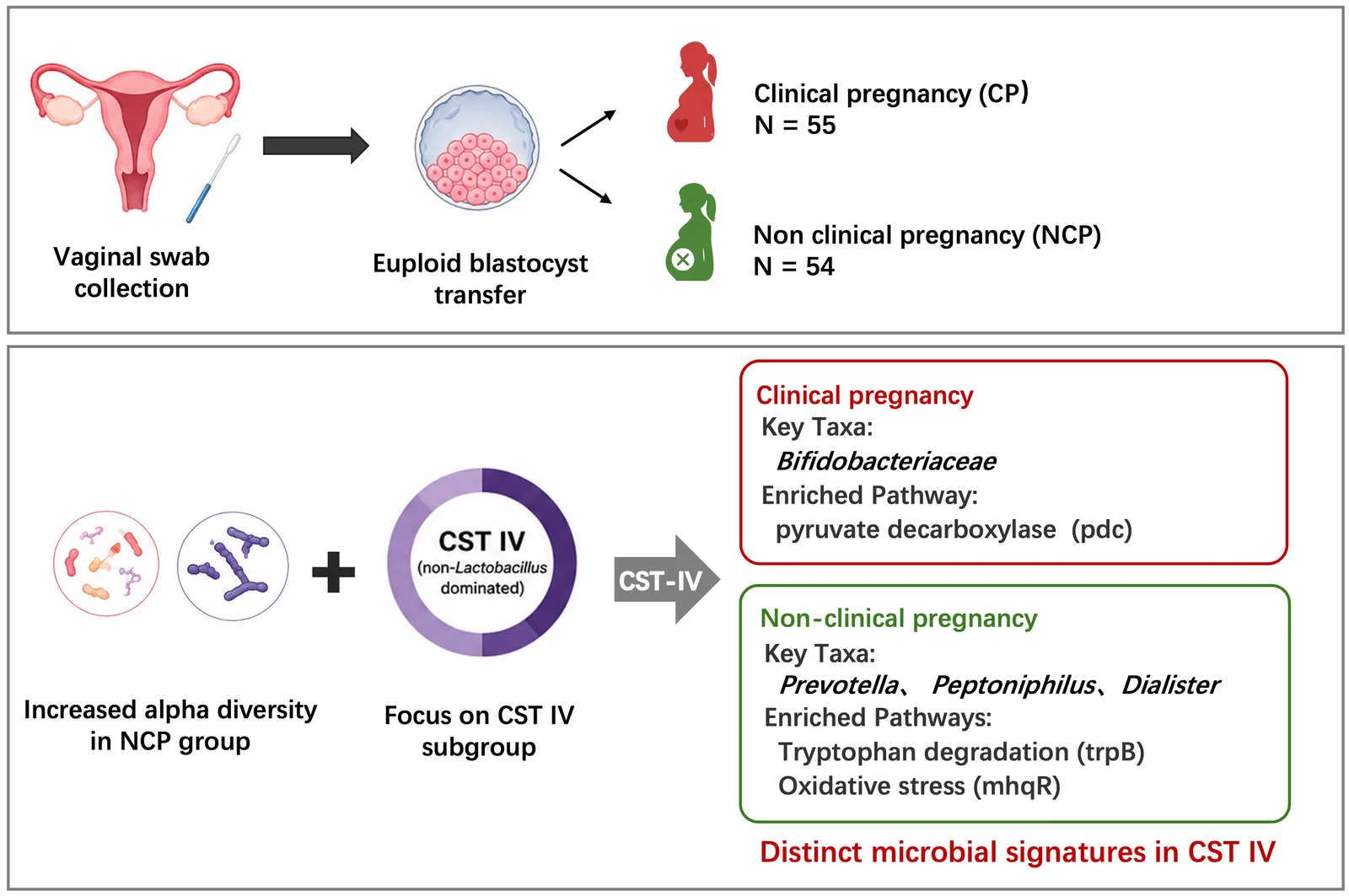
Schematic summary of the study design and key findings. Vaginal swabs were collected prior to euploid blastocyst transfer and stratified by clinical pregnancy (CP, N = 55) versus non-clinical pregnancy (NCP, N = 54). The NCP group exhibited increased alpha diversity. Within the CST IV subgroup, distinct microbial signatures were identified: CP was characterized by enrichment of Bifidobacteriaceae and the pyruvate decarboxylase pathway (pdc), whereas NCP was associated with Prevotella, Peptoniphilus, and Dialister, along with upregulated tryptophan degradation (trpB) and oxidative stress response (mhqR) pathways.

Comparison of vaginal microbiota between the CP and NCP groups revealed significant, albeit modest, differences in community structure. The NCP group exhibited significantly higher species richness quantified by Chao1 and ACE indices, partially consistent with previous reports linking elevated richness to unfavorable reproductive outcomes (7, 16, 48), whereas community evenness assessed by Shannon or Simpson indices remained comparable. This inverse relationship between species richness and pregnancy likelihood was further corroborated by a recent prospective study (49) in which longitudinal sampling across three IVF cycle time points revealed not only significantly lower vaginal microbial diversity in women who achieved clinical pregnancy, but also that the predictive effect of the microbiome was most pronounced before embryo transfer, suggesting a temporal window during which microbial community structure may influence endometrial receptivity. Our subgroup analyses suggest that the microbial signature is more distinct in normal responders, first-time transfers, and ≥5BB embryos, possibly because intense stimulation, repeated interventions, or lower embryo developmental competence might mask native microbiome patterns. The consistency across endometrial thickness subgroups indicates that microbial diversity acts independently of endometrial thickness. Furthermore, the stronger significance in patients without antibiotic exposure reinforces the robustness of our findings by minimizing medication-induced confounding. Beta diversity based on Bray–Curtis dissimilarity and ANOSIM demonstrated a biologically modest yet statistically significant separation (R^2^ = 0.049, *p* = 0.002), which represents an effect size smaller than that reported in previous studies of unselected embryo cohorts (50, 51). We attribute this difference in effect magnitude primarily to embryo quality acting as a critical confounder. Prior studies that included untested embryos reported stronger associations between microbiota composition and pregnancy outcomes, probably because aneuploid embryos are more susceptible to developmental arrest under adverse conditions, such as a dysbiotic microbial environment. By restricting our analysis to transfers of euploid embryos, we demonstrate that the vaginal microbiome exerts a statistically detectable yet quantitatively limited independent effect on implantation outcome, consistent with its role as one of multiple contributing factors rather than a primary determinant.In our study, women with non-*Lactobacillus*-dominant CST-IV achieved pregnancy outcomes comparable to those with *Lactobacillus*-dominant CSTs, contrasting prior reports linking CST-IV to adverse reproductive outcomes (14). Discrepancies in findings among different studies may stem from multiple factors, including substantial differences in the methodological frameworks and resolution of microbial detection across studies (14, 15, 52) as well as the high complexity and inter-individual variability of the vaginal microbiota (53, 54). However, we attribute the primary divergence between our findings and the prior literature to the rigorous control of embryonic aneuploidy, which is the most critical determinant of pregnancy outcomes following embryo transfer. Our result implies that euploid embryos can successfully implant in sub-optimal microbial environments when specific microbial features are present. This interpretation aligns with recent observations by Simon et al. (55) and Wang et al. (56), who showed that broad CST classification offers limited prognostic value. Notably, Wang et al. reported that the *Gardnerella* dominated CST IV-B yielded pregnancy rates comparable to certain *Lactobacillus*-dominant subtypes, further supporting the notion that reproductive competence is not strictly dictated by the absence of *Lactobacillus* but rather by the specific functional configuration of the community.

Our results revealed that CST-IV subgroup encompasses substantial heterogeneity in both taxonomic composition and functional capacity, which jointly shape its reproductive outcomes. This aligns with Ravel et al.’s characterization of CST-IV diversity (10) and recent large-scale Chinese cohort studies identifying 13 Vagitypes and an intermediate microbiome state (57). To mitigate potential statistical bias stemming from the modest CST-IV sample size, we conducted bootstrap sensitivity analysis and validated the major discriminatory genera using real-time PCR quantification. Within CST-IV, successful pregnancy after embryo transfer correlates with *Bifidobacteriaceae* enrichment and fermentative pathways, whereas non-pregnancy associates with *Prevotella*- and *Dialister*-dominant profiles characterized by pro-inflammatory activity and tryptophan catabolism, which has been hypothesized to interfere with the kynurenine pathway essential for maternal-fetal tolerance (58). Mechanistically, although *Lactobacillus* species act as classical protective bacteria by producing lactic acid, *Bifidobacterium* can also generate short-chain fatty acids such as acetate (59) and a range of other bioactive metabolites including folate, riboflavin and bacteriocins (60, 61), which have been implicated in supporting host energy metabolism (62). Notably, the L-lactic acid synthesized by *Bifidobacterium* is more readily metabolized by host cells than the D-lactic acid predominantly produced by *Lactobacillus*, thereby reinforcing vaginal acidity and local homeostasis (63). In contrast, *Prevotella* and *Dialister* have been associated with reduced endometrial receptivity via oxidative stress and inflammatory metabolites (7, 64–66). This functional divergence is corroborated by recent shotgun metagenomic data from Wang et al. (56), who demonstrated that non-pregnant women harbor a significantly higher abundance of antibiotic resistance genes and pathways related to infectious diseases, whereas pregnant women are enriched with beneficial taxa like *L. jensenii*. Collectively, these findings underscore that the functional potential of the microbiome, rather than taxonomy alone, is a critical determinant of implantation success. This challenges the simplistic “beneficial *Lactobacillus* versus harmful CST-IV” dichotomy. Indeed, even within the genus *Lactobacillus*, species are not functionally equivalent, as highlighted by France et al. (67), who reported that *L. crispatus* confers superior protection compared with *L. iners*. Furthermore, CST-IV itself represents a heterogeneous collection of anaerobes rather than a monolithic entity. Taken together, our data tentatively suggest that CST-IV might be better understood not as a uniform risk category, but as a dynamic functional spectrum, in which specific microbial functions outweigh broad taxonomic labels when evaluating reproductive competence (67).

Another emerging concept potentially relevant to our findings is the estrobolome, defined as the collection of microbial genes capable of metabolizing estrogens (68). Several bacterial taxa identified in our cohort, including *Bifidobacterium* and *Prevotella*, encode β-glucuronidases that deconjugate estrogens and thereby modulate their enterohepatic recirculation and systemic bioavailability (6, 69). While estrobolome research in reproduction has focused mainly on the gut microbiome and estrogen-driven conditions such as endometriosis and infertility (68, 70), its role in embryo implantation remains largely unexplored. Our findings align with this emerging framework, which posits endometrial dysbiosis and estrobolome impairment as synergistic contributors to infertility and repeated implantation failure (71). This is consistent with our observation that non-pregnancy outcomes are associated with pro-inflammatory, anaerobe-dominant profiles. Extending this concept, our data suggest that vaginal communities, in addition to gut microbiota, may also shape the local estrogenic milieu at transfer. This is particularly pertinent here, as all patients underwent hormone replacement therapy cycles in which exogenous estrogen drove endometrial preparation; however, since exogenous sex steroids can reshape the vaginal microbiota (72) and hormonal status modifies estrobolome capacity (73), our cross-sectional design cannot distinguish microbial effects on estrogen homeostasis from estrogen-driven selection of specific taxa. Whether the *Bifidobacteriaceae* enrichment observed in pregnant CST-IV patients contributes to a more favorable estrogenic milieu through such mechanisms remains speculative and warrants dedicated metabolomic and hormonal profiling in future studies.

From a clinical perspective, our findings offer preliminary insights regarding the heterogeneity of CST-IV in assisted reproductive technology. Although CST-IV patients including those harboring *Bifidobacterium* have been historically classified as dysbiotic and subjected to unnecessary microbiome-directed interventions, our data indicate that CST-IV status alone may not be uniformly detrimental to euploid embryo transfer outcomes. Given that nearly half of CST-IV women in our cohort achieved live birth, these results raise the hypothesis that non-selective antibiotic administration could potentially deplete beneficial anaerobic taxa including *Bifidobacterium*; however, this remains speculative without interventional validation. This notion parallels the management of chronic endometritis, in which antibiotic treatment followed by histological confirmation of resolution significantly improves IVF outcomes (18), and in which 16S rRNA-based molecular diagnostics have already begun to replace empiric approaches by redefining the condition as a microbiome dysbiosis rather than a simple infection (18). Furthermore, while genus-level biomarkers and predicted functional profiles showed associations with pregnancy outcomes in this exploratory analysis, they do not constitute validated predictive tools. Therefore, rather than supporting immediate precision medicine frameworks, these findings highlight the need for future randomized controlled trials to determine whether tailored, evidence-based interventions targeting specific microbial signatures can improve reproductive outcomes compared to current protocols.

This study possesses notable methodological strengths that enhance the validity of its findings. Critically, it specifically evaluates the impact of the vaginal microbiota on embryo transfer outcomes within the exclusive context of euploid embryo transfers. This deliberate focus effectively controls for the confounding influence of embryonic aneuploidy, a major determinant of implantation failure, thereby isolating the potential contribution of the microbial environment. Furthermore, rigorous inclusion and exclusion criteria were implemented to minimize heterogeneity and mitigate known confounders. By prospectively excluding patients with advanced maternal age, history of recurrent pregnancy loss and recurrent implantation failure, or underlying autoimmune disorders, we established a study population with enhanced clinical homogeneity. Consequently, the observed associations between vaginal microbiota features and pregnancy outcomes are more likely to reflect a genuine biological influence of the microbiome on the receptivity of a euploid embryo.

Several limitations of this study should be noted. First, the small sample size of the CST-IV subgroup poses specific methodological challenges for differential abundance analysis. LEfSe results in such limited cohorts are susceptible to unstable feature selection and inflated effect sizes driven by outliers or sampling variance. To mitigate this concern, we employed a multi-pronged validation strategy: discriminatory taxa were required to meet not only statistical significance but also strong co-occurrence correlations in our network analysis (|r| > 0.6), and the major genera were further confirmed by absolute real-time PCR quantification. Although these measures strengthen the biological plausibility of our findings, the identified taxa should be interpreted as hypothesis-generating candidates rather than validated biomarkers, and confirmation in larger, independent cohorts is warranted. Second, our PICRUSt2-based functional predictions are computational inferences that rely on reference genomes to impute metabolic potential; they cannot capture strain-level genomic variations or actual *in situ* gene expression (74), and require validation through shotgun metagenomics or targeted metabolomics. Third, vaginal microbiota sampling only infers endometrial impacts indirectly. Studies have suggested a strong correlation between vaginal and endometrial microbiota, but the extent of bacterial ascension and local metabolic activity within the uterine cavity remains to be fully elucidated (75, 76). Fourth, the cross-sectional design precludes assessment of vaginal microbiome dynamics across the implantation window. Fifth, while the exclusive use of hormone replacement therapy ensured consistency in endometrial preparation, it limits the generalizability of our findings, precluding the evaluation of how different protocols, such as natural cycles, might influence the association between the vaginal microbiome and transplantation outcomes. Estrogen status itself shapes vaginal microbial composition, as evidenced by the positive correlation between estradiol and *Lactobacillus* abundance in women receiving hormone replacement therapy (6), which may have further homogenized the microbial landscape in our cohort. Furthermore, the use of 97% OTU clustering, while ensuring comparability with prior literature, offers lower taxonomic resolution than ASV-based methods and may obscure strain-level variations. Additionally, although none of the patients had used antibiotics or probiotics during the 1 week preceding embryo transfer, antibiotic or probiotic exposure more than 1 week before embryo transfer was not systematically recorded. Therefore, the potential influence of prior exposure on the vaginal microbiota and the possibility of residual confounding cannot be completely excluded. Finally, as an observational study, our findings reflect statistical associations rather than causal relationships; reverse causation and residual confounding cannot be entirely excluded, and all mechanistic interpretations should be regarded as hypothesis-generating.

Future research should prioritize longitudinal multi-omics studies with larger cohorts to validate the predictive association between vaginal *Bifidobacterium* enrichment and specific metabolic pathways. Meanwhile, interventional trials are needed to determine whether targeted supplementation with *Bifidobacterium* strains can improve implantation rates in women with non-*Lactobacillus*-dominant vaginal microbiomes. Ultimately, developing a functional risk score that integrates taxonomic and metabolic data may replace current CST-based diagnostics, enabling more precise and effective personalized treatments for implantation failure.

## 5 Conclusions

In conclusion, by restricting the analysis to single euploid blastocyst transfers, this study demonstrates that the vaginal microbiome exerts a statistically detectable yet quantitatively limited influence on implantation once embryonic aneuploidy is controlled. Broad CST classification, including CST-IV status, was not associated with clinical pregnancy outcomes, whereas non-pregnant patients exhibited significantly higher microbial richness and a modest but significant shift in community structure. Within the CST-IV subgroup, pregnancy success was characterized by *Bifidobacteriaceae* enrichment, whereas failure was associated with *Prevotella*, *Dialister*, and *Peptoniphilus*. These microbial signatures were supported by co-occurrence network analysis and accompanied by predicted differences in fermentative and pro-inflammatory metabolic pathways. These findings challenge the conventional view that *Lactobacillus* dominance is necessarily linked to reproductive success and suggest that the functional configuration of the microbial community, rather than its taxonomic classification, may be more relevant to the implantation of euploid embryos. Clinically, our results caution against indiscriminate microbiome-directed interventions based solely on CST assignment. Given the observational design and the limited CST-IV sample size, these microbial signatures should be regarded as hypothesis-generating candidates that require validation in larger, longitudinal, multi-omics cohorts before translation into personalized embryo transfer strategies.

## Data Availability

The datasets presented in this study can be found in online repositories. The names of the repository/repositories and accession number(s) can be found in the article/**Supplementary Material**.

## Supplementary material

Supplementary Figure 1 Subgroup analyses of alpha diversity of vaginal microbiota between the NCP and CP groups. Chao1, ACE, Shannon, and Simpson indices were compared within the following subgroups: patients with endometrium ≥10 mm **(A)**, patients with endometrium of 8–10 mm **(B)**, patients with normal ovarian response **(C)**, patients with high ovarian response **(D)**, patients with no antibiotic use in the prior 3 months **(E)**, patients with antibiotic use in the prior 3 months **(F)**, patients with first-time embryo transfers **(G)**, patients with repeated embryo transfers **(H)**, patients with BB-grade embryo transfers **(I)**, patients with BC-grade embryo transfers **(J)**, patients with infertility duration <3 years **(K)**, and patients with infertility duration ≥3 years (**L)**.* indicating significant differences between the two groups.

Supplementary Figure 2 LEfSe identification of discriminatory vaginal microbial taxa within stratified CST IV patients. Stratified LEfSe analyses comparing CP and NCP groups were performed within the following subgroups: patients with endometrium of 8–10 mm **(A)**, patients with endometrium ≥10 mm **(B)**, patients with normal ovarian response **(C)**, patients with high ovarian response **(D)**, patients with no antibiotic use in the prior 3 months **(E)**, patients with first-time embryo transfers **(F)**, patients with BB-grade embryo transfers **(G)**, patients with BC-grade embryo transfers **(H)**, patients with infertility duration <3 years **(I)**, and patients with infertility duration ≥3 years **(J)**. Taxa with LDA scores exceeding the preset threshold are displayed. Valid LEfSe comparison was not feasible for the subgroups with antibiotic use in the prior 3 months and patients with repeated embryo transfers.

Supplementary Figure 3 Real-time PCR validation of differentially abundant taxa identified by LEfSe analysis in the CST-IV subgroup. Relative expression levels of **(A)** Prevotella bivia, **(B)** Dialister micraerophilus, and **(C)** Bifidobacterium bifidum were quantified by real-time PCR in the NCP and CP groups. Data are presented as mean ± SEM. * indicating significant differences between the two groups.
